# The (Biological or Cultural) Essence of Essentialism: Implications for Policy Support among Dominant and Subordinated Groups

**DOI:** 10.3389/fpsyg.2017.00900

**Published:** 2017-05-30

**Authors:** Nur Soylu Yalcinkaya, Sara Estrada-Villalta, Glenn Adams

**Affiliations:** Department of Psychology, University of KansasLawrence, KS, United States

**Keywords:** cultural essentialism, strategic essentialism, diversity, affirmative action, cultural inclusion, immigration policy

## Abstract

Most research links (racial) essentialism to negative intergroup outcomes. We propose that this conclusion reflects both a narrow conceptual focus on biological/genetic essence and a narrow research focus from the perspective of racially dominant groups. We distinguished between beliefs in biological and cultural essences, and we investigated the implications of this distinction for support of social justice policies (e.g., affirmative action) among people with dominant (White) and subordinated (e.g., Black, Latino) racial identities in the United States. Whereas, endorsement of biological essentialism may have similarly negative implications for social justice policies across racial categories, we investigated the hypothesis that endorsement of cultural essentialism would have different implications across racial categories. In Studies 1a and 1b, we assessed the properties of a cultural essentialism measure we developed using two samples with different racial/ethnic compositions. In Study 2, we collected data from 170 participants using an online questionnaire to test the implications of essentialist beliefs for policy support. Consistent with previous research, we found that belief in biological essentialism was negatively related to policy support for participants from both dominant and subordinated categories. In contrast, the relationship between cultural essentialism and policy support varied across identity categories in the hypothesized way: negative for participants from the dominant category but positive for participants from subordinated categories. Results suggest that cultural essentialism may provide a way of identification that subordinated communities use to mobilize support for social justice.

## Introduction

Essentialism refers to a lay belief in the existence of underlying natures that constitute and differentiate social categories (e.g., Haslam et al., 2000, 2002; Bastian and Haslam, 2006). It entails the tendency to understand social categories as expressions of discrete, fixed, natural, uniform, and defining characteristics that are shared by all members, and are informative about them (Haslam et al., 2000, 2002; Bastian and Haslam, 2006). Endorsement of essentialist beliefs has commonly been associated with negative outcomes, such as legitimization of existing social hierarchies (Jost et al., 2004; Keller, 2005; Williams and Eberhardt, 2008; Morton et al., 2009b), acceptance of racial inequality (Williams and Eberhardt, 2008), and anti-Black prejudice among White Americans (Condit et al., 2004; Keller, 2005; Jayaratne et al., 2006; Andreychik and Gill, 2014).

Is racial essentialism necessarily negative and oppressive? We propose that this standard verdict about essentialism results from two features of most research. First, investigations of racial essentialism typically understand the defining characteristics of an identity category (i.e., race) in terms of a biological essence. However, people may also understand racial categories in terms of cultural characteristics, rather than purely biological or genetic characteristics. The implications of such beliefs in a cultural essence may be more open to various identity-relevant interpretations. Second, most of the research that implicates racial essentialist beliefs in the reproduction of social inequality has exclusively considered the perspective of people in racially dominant groups. However, people in marginalized or oppressed communities might “strategically use a positive essentialism” (Spivak, 1988, p. 13) as a basis for collective identity and political action. We set out to expand research on racial essentialism by (1) providing a more nuanced conceptualization of essentialism as based on beliefs in different kinds of essences (biological versus cultural), and (2) investigating the implications of biological and cultural forms of essentialism for policy support among dominant and subordinated racial groups.

### The essence of essentialism

“Essence” is an elusive concept; it is difficult to define precisely what comprises the imagined essence of an identity category (Prentice and Miller, 2007). The literature commonly defines racial essentialism as a belief in a genetic or biological essence that defines all members of a racial category (e.g., Race Conceptions Scale; Williams and Eberhardt, 2008; cf. Andreychik and Gill, 2014). Researchers have identified biological basis as one of the dimensions of essentialism (Bastian and Haslam, 2006). However, the imagined essence of a racial group need not be biological; people also define racial groups in terms of particular cultural characteristics, or conflate race and culture (e.g., No et al., 2008; Hong et al., 2009; Morning, 2009). Relatedly, essentialist beliefs about race can have implications for perceived cultural differences and cultural identification (No et al., 2008). It is possible for individuals to understand racial categories in terms of essential cultural features. *Cultural essentialism* is the idea that “[p]eople are …more or less passive carriers of their culture, whereby their attitudes, beliefs and achievements are supposed to reflect typical cultural patterns” (Verkuyten, 2003, p. 385). Applied to racial identity, cultural essentialism is the belief that racial categories are associated with distinct, fixed, and stable cultural patterns (e.g., values, beliefs, practices, and lifestyles); these fixed cultural patterns definitively and permanently shape the psychological characteristics of individuals within a racial group, and differentiate them from members of other racial groups. Cultural and biological forms of racial essentialism share the idea that differences between racial groups are determined by a fixed and uniform essence that resides within and defines all members of each racial group. However, they differ in their understanding of the nature of this essence. Both forms of essentialism may coexist; indeed, many people perceive race as having both biological and cultural foundations (Morning, 2011; Byrd and Ray, 2015).

Research suggests that people strategically endorse or downplay essentialism depending on identity concerns (Morton et al., 2009a). Endorsement of (biological) essentialism is typically greater among people who identify with dominant identity categories (e.g., high social class rank; Kraus and Keltner, 2013). For instance, endorsement of gender essentialism is stronger among men than women, especially when male social dominance is under threat (Morton et al., 2009b). Despite this tendency of dominant group members to endorse essentialism, White respondents—especially those who report high racial prejudice—endorse essentialist ideas about their racial group less when they perceive that White identity might cause them to be excluded from some benefit (Morton et al., 2009a).

Results of a focus group study in the Netherlands suggest that people can also use cultural forms of essentialism to advance collective interests (Verkuyten, 2003). Specifically, both ethnic minority and ethnic Dutch participants expressed cultural essentialist ideas at times, but they did so for different ends. For instance, ethnic minority participants expressed cultural essentialist ideas to argue against assimilationist policies, claiming that they could not possibly “become just like the Dutch” in order to fully integrate into Dutch society (Verkuyten, 2003, p. 381). Ethnic Dutch participants expressed cultural essentialist ideas—specifically, the belief that a person absorbs the ethnic culture within which he/she is raised, with the implication that Dutch and other ethnic groups are fundamentally different types of person—to argue for restrictions on entry of immigrants into the country (Verkuyten, 2003). However, ethnic Dutch participants also discussed ethnic identity in non-essentialist terms, particularly to argue that minority groups *can*—and should—assimilate to Dutch culture (Verkuyten, 2003).

### Turning the lens: research from the perspective of marginalized groups

Knowledge in mainstream psychological science has a disproportionate basis in research from the perspective of people from dominant groups (e.g., Arnett, 2008; Henrich et al., 2010). In combination with the idea that people deploy essentialism in a strategic fashion, this fact suggests a reason why researchers have typically observed a negative association between essentialism and egalitarian social policy (for exceptions to research focusing exclusively on dominant viewpoints, see No et al., 2008 and Williams and Eberhardt, 2008). People from racially dominant groups tend to interpret and deploy essentialism in ways that reproduce the unjust status quo. What happens if we decolonize scientific gaze and consider the issue of essentialism from the standpoint of marginalized or racially subordinated identity positions?

Research suggests that people from subordinated categories may often resist essentialist understandings; for example, when they aim to overcome discrimination by challenging constructions of an identity category as a homogeneous entity (Verkuyten and Brug, 2004). This resistance may be particularly true of biological essentialism of race. Historically, people have developed and deployed arguments about genetic differences between racial groups to argue that certain groups of people are naturally inferior in ability, fitness, or intelligence compared to dominant groups, thereby legitimizing forms of racial oppression such as colonialism, slavery, and segregation (Davis, 1991; Smedley and Smedley, 2005; Dar-Nimrod and Heine, 2011). Given the historical role of beliefs in biologically determined racial differences in justifying racial oppression, endorsement of these beliefs is likely to be incompatible with support for social justice policies, especially those designed to address racial inequality by providing academic and occupational opportunities to people from marginalized communities (e.g., various forms of affirmative action). Endorsement of biological essentialism is likely to be weaker among people from subordinated than dominant identity categories, given the implications of such beliefs for the assumed inferiority of subordinated groups (Mahalingam, 2003). Since it is unlikely for genetic essentialist beliefs to be deployed as grounds for collective identification or advancement of group interests among subordinated groups, one can hypothesize (H1) that endorsement of biological essentialism in the context of racial categories will have the same negative relationship with support for social justice policies among people from dominant and subordinated categories.

Unlike biological essentialism, beliefs in cultural essentialism may have more divergent implications for endorsement of social justice policy. Similar to the case of biological essentialism, among people from dominant categories, endorsement of cultural essentialism may imply a belief that racial inequality reflects the essential superiority of their own cultural practices and essential inferiority of other practices. This belief may undergird support for anti-egalitarian policies, especially those designed to protect against symbolic threat to the dominant cultural order (e.g., restrictive immigration; on the effect of perceived symbolic threat on negative immigrant attitudes, see Stephan et al., 2005; McLaren and Johnson, 2007). However, for people from racially subordinated communities, endorsement of cultural essentialism may not have the same implications. Although one can certainly observe patterns of *colonial mentality* (David and Okazaki, 2006) whereby people from subordinated communities adopt dominant understandings of essential inferiority, an enduring contribution of anti-colonial and ethnic nationalist movements is to promote viewpoints that emphasize the positive distinctiveness of subordinated community identity (Fanon, 1963). These alternatives to dominant discourse promote “models of identification” (Martín-Baró, 1994, p. 30) that constitute a foundation for collective action and resistance (Simon and Klandermans, 2001). Consistent with this idea, Mahalingam (2003) suggests that people from racially subordinated groups are likely to endorse social or non-biological varieties of essentialism to a greater extent than people from dominant groups. Moreover, research among people from marginalized ethnic minorities in the Netherlands indicates that endorsement of cultural essentialist beliefs with regards to one's own ethnic group is associated with assertion of identity, advocacy for recognition of cultural rights, and greater support for multiculturalism (Verkuyten, 2003; Verkuyten and Brug, 2004). In summary, previous evidence for identity-enhancing interpretations of cultural essentialism suggests a moderation hypothesis (H2), whereby endorsement of cultural essentialism has divergent implications for different identity categories: negatively related to support for social justice policies among people from dominant racial categories, but positively related to support for social justice policies among people from subordinated racial categories^1^.

### Studies 1a and 1b: scale development

Before testing our hypotheses regarding the relationships between the two forms of essentialism and policy support among members of dominant and subordinated racial groups, we set out to examine the reliability and construct validity of our measure of cultural essentialism. We developed 12 items to measure beliefs in cultural essentialism with respect to racial categories. We based these items on existing definitions of cultural essentialism in the literature (e.g., Verkuyten, 2003) to capture the belief that each racial group has distinct and uniform cultural patterns, which shape the individuals that identify with the group and determine the kind of person they are. We included items in our measure that tap various aspects of cultural patterns, such as values, beliefs, lifestyle and behaviors. In Studies 1a and 1b, we examined the properties of our measure and how it relates to (and is distinct from) relevant measures such as genetic essentialism, implicit theories of race, and social dominance orientation, on two samples with different racial/ethnic compositions.

## Study 1A

### Methods

#### Participants and procedure

We recruited 196 participants online through our university pool^2^. We excluded 23 participants from our analyses because they failed two or more of our attention-check items. The final sample composed of 173 participants (39.3% female, 35.3% male, 25% missing^3^; 69.4% White, 5.2% Latino, 3.5% Asian, 2.9% African American, 8.1% mixed race and other, 11% missing). Participants completed a set of questionnaires and received partial course credit.

#### Measures

Participants completed the cultural essentialism measure (e.g., “Every racial group has a distinctive, defining culture of their own” and “People raised in different racial communities learn to behave, think and talk in ways defining of their racial group,” α = 0.85), the Race Conceptions Scale (e.g., “The physical features of different racial groups haven't really changed much over the centuries,” α = 0.76, Williams and Eberhardt, 2008), the Implicit Person Theory measure (e.g., “Everyone is a certain kind of person, and there is not much that they can do to really change that,” α = 0.87, Chiu et al., 1997), Internal and External Motivation to Respond Without Prejudice Scales (e.g., “I attempt to act in non-prejudiced ways toward Black people because it is personally important to me” and “Because of today's politically correct standards I try to appear non-prejudiced toward Black people,” αs = 0.87 and 0.80, respectively, Plant and Devine, 1998), Social Dominance Orientation Scale (e.g., “Some groups of people are simply inferior to other groups,” α = 0.95, Pratto et al., 1994), a measure of racial identification (e.g., “I feel strong ties to other members of my racial/ethnic group,” α = 0.73, Cameron, 2004), and the Marlowe–Crowne Social Desirability Scale (e.g., “I have never deliberately said something that hurt someone's feelings,” Crowne and Marlowe, 1960). Participants rated all items using 7-point Likert-type scales, ranging from 1 (Strongly Agree) to 7 (Strongly Disagree), except for the Social Dominance Orientation Scale, for which the response options ranged from 1 (Very Negative) to 7 (Very Positive). Following the original scoring of the Social Desirability Scale, we recoded the items as true or false (0 or 1, depending on the item) before calculation of average scores.

### Results

A factor analysis with maximum likelihood extraction and direct oblimin rotation on the 12 items of our cultural essentialism measure yielded two factors. The two factors did not map onto a clear theoretical distinction. The scale had high reliability with all items included (α = 0.85). We therefore proceeded to calculate a cultural essentialism score composed of all 12 items. Scores on the cultural essentialism measure showed a fairly normal distribution (skewness = 0.031, *SE* = 0.19; kurtosis = 1.20, *SE* = 0.37). The mean was above the midpoint of the rating scale (*M* = 4.45, *SD* = 0.79). Women (*M* = 4.52, *SD* = 0.84) and men (*M* = 4.50, *SD* = 0.83) did not differ in their scores on the measure, *t*_(124)_ = 0.159, *p* = 0.9. Participants who identified with racial categories other than White scored slightly higher on the cultural essentialism measure (*M* = 4.57, *SD* = 0.62) than participants who identified as White (*M* = 4.41, *SD* = 0.86). This difference was not significant, *t*_(151)_ = −1.041, *p* = 0.3; however, we had very few non-White participants (*N* = 34) compared to White participants (*N* = 119) in our sample^4^.

Participants' scores on the cultural essentialism measure positively correlated with their scores on the Race Conceptions Scale developed by Williams and Eberhardt (2008) as a measure of beliefs in the genetically determined, fixed, and stable nature of racial categories (*r* = 0.36, *p* = 0.00). This finding, which suggests that participants who tend to essentialize race in genetic terms also tend to believe in cultural essences underlying racial groups, is in keeping with research by Morning (2011) showing that people think of race as having both genetic and cultural bases. However, the correlation is moderate in strength, suggesting that the two constructs are distinct. Participants' scores on the cultural essentialism measure also positively correlated with the Implicit Person Theory measure (*r* = 0.39, *p* = 0.00), which taps the idea of fixedness of personality. Cultural essentialism scores correlated positively with the External Motivation to Respond Without Prejudice Scale (*r* = 0.19, *p* = 0.01), but negatively with the Internal Motivation to Respond Without Prejudice Scale (*r* = −0.26, *p* = 0.001). This suggests that participants who hold cultural essentialist beliefs about race are likely to be concerned about not appearing prejudiced, rather than following internal norms of being non-prejudiced. Participants' scores on the cultural essentialism measure positively correlated with racial identification (*r* = 0.18, *p* = 0.01) and social dominance orientation (*r* = 0.33, *p* = 0.00). Finally, scores on the cultural essentialism measure did not correlate with the Social Desirability Scale (*r* = −0.09, *p* = 0.25).

## Study 1B

In Study 1a, our sample consisted of mostly White university students. In keeping with the aim and hypotheses of our research, in Study 1b, we specifically targeted non-White participants to investigate whether our measure of cultural essentialism had a similar structure and construct validity among participants from subordinated racial groups. We expected the cultural essentialism measure to show the same patterns of relationship with the scales that we used in Study 1a, with the exception of social dominance orientation: in line with H2, we did not expect a positive relationship between cultural essentialism and social dominance orientation among our non-White sample.

### Methods

#### Participants and procedure

We recruited 86 participants online through Amazon's Mechanical Turk website, targeting participants who identify with racial categories other than White. We excluded 3 participants from our analyses because they failed two or more of our attention-check items. The final sample composed of 83 participants (44% female, 56% male; 50% African American, 18.3% Latino, 13.4% Asian, 12.2 Native American, 6% mixed race and other, 1 missing). Participants completed a set of questionnaires and received monetary compensation.

#### Measures

Participants completed the same set of measures as in Study 1a: the cultural essentialism measure (α = 0.88), the Race Conceptions Scale (α = 0.86, Williams and Eberhardt, 2008), Implicit Person Theory measure (α = 0.90, Chiu et al., 1997), Internal and External Motivation to Respond Without Prejudice Scales (e.g., αs = 0.89 and 0.93, respectively, Plant and Devine, 1998), Social Dominance Orientation Scale (α = 0.98, Pratto et al., 1994), Cameron's (2004) measure of racial identification (α = 0.84), and the Marlowe–Crowne Social Desirability Scale (Crowne and Marlowe, 1960).

### Results

A factor analysis with maximum likelihood extraction and direct oblimin rotation on the 12 items of our cultural essentialism measure yielded 3 factors. The factors did not map onto a clear theoretical distinction. The scale had high reliability with all items included (α = 0.88). As in Study 1a, we proceeded to calculate a cultural essentialism score composed of all 12 items. Scores on the cultural essentialism measure showed a fairly normal distribution (skewness = −0.20, *SE* = 0.26; kurtosis = 1.34, *SE* = 0.52). The mean was above the midpoint of the rating scale (*M* = 4.50, *SD* = 0.95). Women (*M* = 4.55, *SD* = 1.10) and men (*M* = 4.46, *SD* = 0.83) did not differ in their scores on the cultural essentialism measure, *t*_(82)_ = −0.43, *p* = 0.7.

As in Study 1a, participants' scores on the cultural essentialism measure positively and moderately correlated with their scores on the Race Conceptions Scale (*r* = 0.47, *p* = 0.00). Again, participants' scores on the cultural essentialism measure positively correlated with the Implicit Person Theory measure (*r* = 0.36, *p* = 0.00), and the External Motivation to Respond Without Prejudice Scale (*r* = 0.17, *p* = 0.12), but negatively with the Internal Motivation to Respond Without Prejudice Scale (*r* = −0.15, *p* = 0.17), although the latter correlations were not significant. Cultural essentialism scores positively correlated with racial identification (*r* = 0.24, *p* = 0.03). However, unlike in Study 1a, cultural essentialism did not show a significant correlation with social dominance orientation (*r* = 0.10, *p* = 0.38), suggesting that among participants who identify with racial categories other than the dominant White category, those who endorse cultural essentialist beliefs do not necessarily endorse social hierarchies. This finding is consistent with our hypothesis that cultural essentialist beliefs are not necessarily incompatible with a social justice orientation among people with subordinated identities. Finally, endorsement of cultural essentialist beliefs was not related with concerns about social desirability (*r* = −0.05, *p* = 0.65).

### Discussion

Studies 1a and 1b suggest that the cultural essentialism measure we developed has sufficient internal consistency, and shows construct validity, across two samples (Table 1). In our mostly White sample (Study 1a) and non-White sample (Study 1b), cultural essentialism showed similar relationships with genetic essentialism, implicit person theory, external, and internal motivation to respond without prejudice, and racial identification. However, in our non-White sample (Study 1b), it did not relate to social dominance orientation. This is in keeping with our hypothesis (H2) regarding possible differences in the implications of the endorsement of these beliefs across racial categories, which we investigate directly in Study 2.

**Table 1:** Correlations between cultural essentialism and other variables across samples.

**Variables**	**Cultural essentialism**	
	**Study 1a (*n* = 165)**	**Study 1b (*n* = 82)**
Genetic Essentialism	0.36^**^	0.47^**^
Implicit Person Theory	0.39^**^	0.36^**^
Internal Motivation to Respond without Prejudice	−0.26^**^	−0.15
External Motivation to Respond without Prejudice	0.19^*^	0.17
Racial Identification	0.18^*^	0.24^*^
Social Dominance Orientation	0.33^**^	0.10
Social Desirability	−0.09	−0.05

* *p < 0.05*,

** *p < 0.01*.

## Study 2

We conducted an online survey study to test our hypotheses on the relationship between cultural essentialism and policy support. Specifically, we assessed the relationship between cultural and biological forms of essentialism and support for social justice policies (affirmative action, cultural inclusion, and demilitarized border security) among racially dominant White participants and participants from subordinated, non-White racial/ethnic groups in the United States (US).

### Methods

#### Participants and procedure

We recruited 170 participants online through Mechanical Turk [50.6% male, *M*_age_ = 36.1 (*SD* = 12.9), 56.5% White, 23.5% African American, 10.6% Latino, 4.1% Native American, 3.5% mixed race and other].^5^ Participants completed a set of questionnaires and received monetary compensation.

#### Measures

All items were measured using 7-point Likert-type scales, ranging from 1 (Strongly Agree) to 7 (Strongly Disagree). A complete list of items appears in the Supplementary Materials.

##### Biological essentialism

As our aim in this study was to distinguish the correlates of beliefs in cultural essences from beliefs in biological essences of race, we used items that tap the genetic/biological component of essentialism directly as our measure of biological essentialism, as opposed to the Race Conceptions Scale (Williams and Eberhardt, 2008) that taps multiple components of essentialism. We adapted four items from the *Belief in Genetic Determinism* scale (Keller, 2005) to measure the extent to which people believe genetic factors definitively shape members of a racial category (e.g., “I think that differences between people of different races in behavior and personality are largely determined by genetic predisposition,” α = 0.91).

##### Cultural essentialism

We used our 12-item scale to measure cultural essentialism (α = 0.88).

##### Policy support

We created three measures of policy support. Eight items assessed support for *affirmative action* for racial minorities in the US (e.g., “Companies should implement quotas to make sure racial minorities are represented in their organization,” α = 0.88). Five items assessed support for *cultural inclusion* of diverse populations within the US (e.g., “American businesses, such as supermarkets, should provide the option to print receipts in multiple languages,” α = 0.78). Measures of support for affirmative action and cultural inclusion focus on targets within the US society; these policies may have direct implications for the participants who are members of subordinated racial groups in the US themselves. We also included a measure in our study that focuses on policies regarding targets outside the US society who intend to enter its geographic space. We used five items assessed support for *demilitarization of US borders*, (e.g., “The U.S. government should reduce the number of Border Patrol traffic checkpoints throughout the country,” α = 0.87). This policy may or may not have direct implications for our participants who identify with subordinated racial groups. We included all three measures to explore whether essentialist beliefs might relate particularly strongly to support for policies that are directly relevant for the participants own racial/ethnic group (i.e., the former two policies for subordinated group members).

##### Demographics

Finally, participants indicated their age, gender, and racial category identification in an open-ended format.

### Results

We classified participants who reported identification as White, Caucasian, or European as “dominant” (*N* = 94), and coded them as “0.” We classified participants who reported identification with any other racial category as “subordinated” and coded them as “1” (*N* = 76).^6^ We use this binary race variable in all of the analyses that follow. Bivariate correlations between key variables and means and standard deviations as a function of racial identity category appear in Tables 2, 3.

**Table 2:** Correlations among key variables and means (and standard deviations) for the dominant racial category.

	**Biological essentialism**	**Cultural essentialism**	**Affirmative action**	**Cultural inclusion**	**Means (Standard deviations)**
Biological Essentialism	–				3.07 (1.54)
Cultural Essentialism	0.509^**^	–			4.58 (0.96)
Affirmative Action	−0.341^**^	−0.406^**^	–		3.79 (1.15)
Cultural Inclusion	−0.550^**^	−0.469^**^	0.495^**^	–	4.74 (1.35)
Demilitarized Borders	−0.398^**^	−0.458^**^	0.570^**^	0.683^**^	3.63 (1.58)

*p < 0.05*,

** *p < 0.01*.

**Table 3:** Correlations among key variables and means (and standard deviations) for the subordinated racial category.

	**Biological essentialism**	**Cultural essentialism**	**Affirmative action**	**Cultural inclusion**	**Means (Standard deviations)**
Biological Essentialism	–				3.44 (1.52)
Cultural Essentialism	0.323^**^	–			4.59 (0.88)
Affirmative Action	0.126	0.250*	–		4.19 (1.07)
Cultural Inclusion	−0.431^**^	0.077	0.229^**^	–	5.05 (1.57)
Demilitarized Borders	−0.268*	−0.047	0.190	0.438^**^	3.90 (1.60)

We conducted three-step hierarchical regression analyses with support for social justice policy measures as outcomes. Because participants in the dominant category were older (*M* = 40.05, *SD* = 13.9) than participants in the subordinated category (*M* = 31.22, *SD* = 9.5), *t*_(168)_ = 4.70, *p* = 0.00), we entered age in the first step of the analyses. In the second step, we entered conceptual predictors: biological essentialism, cultural essentialism, and racial category. In the third step, we entered two-way interactions of racial category with each of the two essentialism variables. These interaction terms assessed the moderating effect of racial category on the relationship between different forms of essentialism and each outcome variable.

#### Affirmative action support

**Figure 1:**
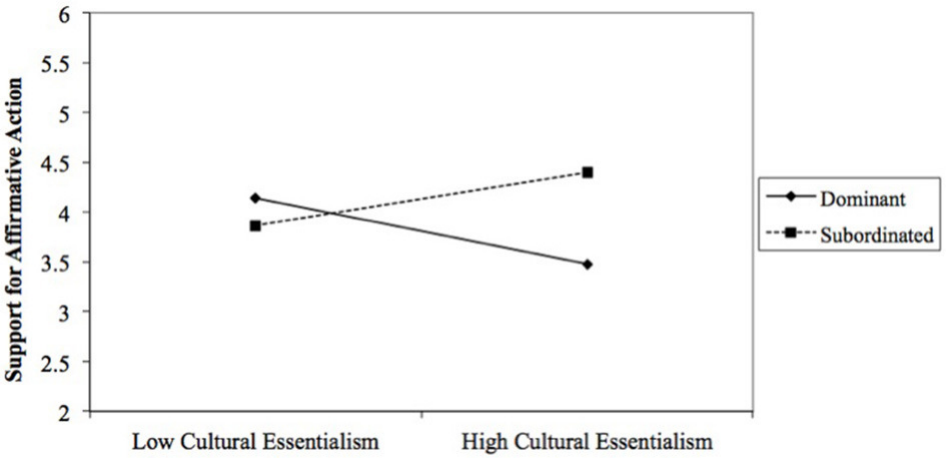
The Interaction between cultural essentialism and racial category on affirmative action support. Other variables included in the model are age and biological essentialism.

The final model predicting affirmative action support was significant, *F*_(6, 163)_ = 5.993, *p* = 0.00, *R*^2^ = 0.18 (Table 4). Consistent with the identity moderation hypothesis (H2), results revealed a significant interaction of racial category and cultural essentialism, *b* = 0.65, *SE* = 0.19, *t*_(163)_ = 3.32, *p* = 0.001, CI [0.26, 1.03]. A probe of this interaction (Figure 1) revealed the hypothesized pattern.^7^ Whereas the relationship between cultural essentialism and support for affirmative action was negative among participants in the dominant category (*b* = −0.36, *SE* = 0.13, *t* = −2.77, *p* = 0.00), it was positive among participants in the subordinated category (*b* = 0.29, *SE* = 0.14, *t* = 1.98, *p* = 0.04). We also deconstructed the interaction to test the racial category difference at low and high levels of cultural essentialism. The racial category difference in support for affirmative action was evident only among participants who scored one standard deviation above the mean on cultural essentialism (*b* = 0.92, *SE* = 0.25, *t* = 3.67, *p* = 0.00), but not those who scored one standard deviation below the mean (*b* = −0.28, *SE* = 0.25, *t* = −1.11, *p* = 0.27). Based on regions of significance tests, the racial category difference emerged at moderate to high (>0.3 SDs) levels of cultural essentialism.

**Table 4:** Hierarchical regression for affirmative action support.

**Total Sample (*N* = 170)**	**Model 1**	**Model 2**	**Model 3**
Variables	β	β	β
Age	−0.17^*^	−0.12	−0.13
Biological Essentialism (Bio Ess)	–	−0.11	−0.20
Cultural Essentialism (Cul Ess)	–	−0.09	−0.29^**^
Racial Category (Race)	–	0.15	0.14
Bio Ess^*^Race	–	–	0.17
Cul Ess^*^Race	–	–	0.33^**^
R^2^	0.03	0.07	0.18

*Numerical entries represent standardized regression coefficients*.

* p < 0.05;

** *p < 0.01*.

The corresponding interaction of racial category and endorsement of biological essentialism was not significant. Independent of racial category, the hypothesized (H1) negative relationship between biological essentialism and affirmative action support approached conventional levels of statistical significance, *b* = −0.15, *SE* = 0.08, *t*_(163)_ = −1.84, *p* = 0.067, CI [−0.31, 0.01].

#### Support for cultural inclusion

**Figure 2:**
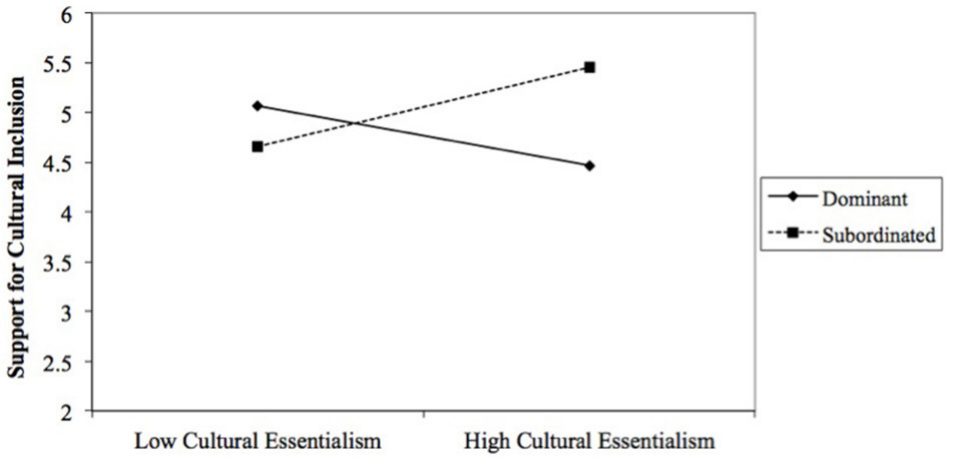
The interaction between cultural essentialism and racial category on support for cultural inclusion. Other variables included in the model are age and biological essentialism.

The final model predicting support for cultural inclusion was significant, *F*_(6, 163)_ = 13.836, *p* < 0.001, *R*^2^ = 0.34 (Table 5). Consistent with the identity moderation hypothesis (H2), results revealed a significant interaction of racial category and cultural essentialism, *b* = 0.76, *SE* = 0.22, *t* = 3.36, *p* = 0.001, CI [0.31, 1.20]. A probe of this interaction (Figure 2) again revealed the hypothesized pattern. Whereas the relationship between cultural essentialism and support for cultural inclusion was negative among participants in the dominant category (*b* = −0.32, *SE* = *0.1*5, *t* = −2.14, *p* = 0.03), it was positive among participants in the subordinated category (*b* = 0.43, *SE* = 0.17, *t* = 2.59, *p* = 0.01). The racial category difference was evident only among participants who scored one standard deviation above the mean on cultural essentialism (*b* = 0.98, *SE* = 0.29, *t* = 3.42, *p* = 0.00), but not those who scored one standard deviation below the mean (*b* = −0.41, *SE* = 0.29, *t* = −1.43, *p* = 0.15). Based on regions of significance tests, the racial category difference emerged among those who scored moderate to high (>0.15 SDs) on the measure of cultural essentialism.

**Table 5:** Hierarchical regression on support for cultural inclusion.

**Total Sample (*N* = 170)**	**Model 1**	**Model 2**	**Model 3**
Variables	β	β	β
Age	−0.20^**^	−0.21^**^	−0.21^**^
Biological Essentialism (Bio Ess)	–	−0.50^**^	−0.42^**^
Cultural Essentialism (Cul Ess)	–	0.01	−0.21^*^
Racial Category (Race)	–	0.10	0.10
Bio Ess^*^Race	–	–	−0.08
Cul Ess^*^Race	–	–	0.31^**^
R^2^	0.04	0.29	0.34

*Numerical entries represent standardized regression coefficients*.

* p < 0.05;

** *p < 0.01*.

The corresponding interaction of racial category and endorsement of biological essentialism was not significant. Consistent with hypothesis H1 and independent of racial category, biological essentialism was a significant negative predictor of support for cultural inclusion, *b* = −0.40, *SE* = 0.09, *t* = −4.21, *p* = 0.00, CI [−0.59, −0.21].

#### Support for demilitarized borders

**Figure 3:**
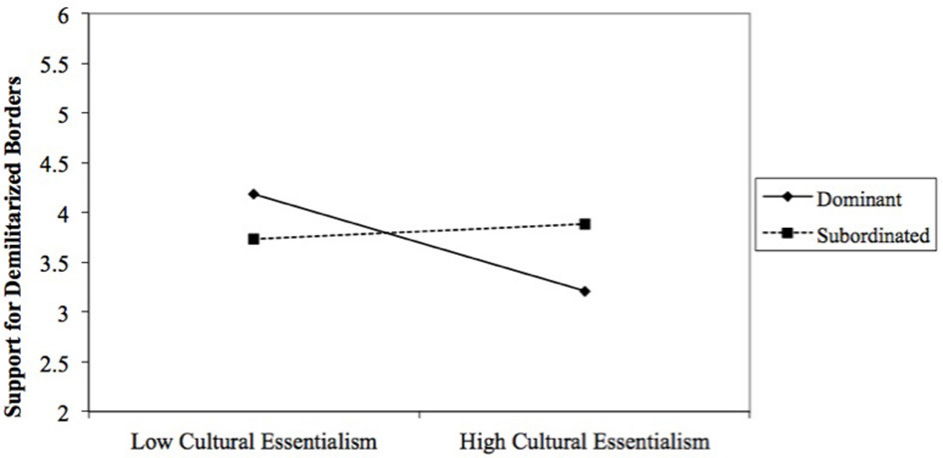
The interaction between cultural essentialism and racial category on support for demilitarized borders. Other variables included in the model are age and biological essentialism.

The final model predicting support for demilitarization of the borders was significant, *F*_(6, 163)_ = 7.645, *p* < 0.001, *R*^2^ = 0.22 (Table 6). Consistent with the identity moderation hypothesis (H2), results revealed a significant interaction of racial category and cultural essentialism, *b* = 0.61, *SE* = 0.27, *t* = 2.28, *p* = 0.023, CI [0.08, 1.1]. A probe of this interaction (Figure 3) revealed that the relationship between cultural essentialism and support for reduced militarization of border security was negative among participants in the dominant category (*b* = −0.53, *SE* = 0.18, *t* = −2.95, *p* = 0.003), but not among participants in the subordinated category (*b* = 0.08, *SE* = 0.20, *t* = 0.42, *p* = 0.67). The racial category difference in support for demilitarized borders was evident only among participants who scored one standard deviation above the mean on cultural essentialism (*b* = 0.67, *SE* = 0.34, *t* = 1.95, *p* = 0.05), but not those who scored one standard deviation below the mean (*b* = −0.45, *SE* = 0.34, *t* = −1.34, *p* = 0.18). Based on regions of significance tests, the racial category difference emerged only among those who scored higher (>0.95 SDs) on the measure of cultural essentialism.

**Table 6:** Hierarchical regression on support for demilitarized borders.

**Total Sample (*N* = 170)**	**Model 1**	**Model 2**	**Model 3**
Variables	β	β	β
Age	−0.22^**^	−0.23^**^	−0.23^**^
Biological Essentialism (Bio Ess)	–	−0.29^**^	−0.26^*^
Cultural Essentialism (Cul Ess)	–	−0.15	−0.31^**^
Racial Category (Race)	–	0.03	0.03
Bio Ess^*^Race	–	–	−0.02
Cul Ess^*^Race	–	–	0.23^*^
R^2^	0.05	0.19	0.22

*Numerical entries represent standardized regression coefficients*.

* p < 0.05;

** *p < 0.01*.

The corresponding interaction of racial category and endorsement of biological essentialism was not significant. Consistent with hypothesis H1 and independent of racial category, biological essentialism was a significant negative predictor of support for demilitarization of the border, *b* = −0.27, *SE* = 0.11, *t* = −2.37, *p* = 0.02, CI [−0.49, −0.04].

### Discussion

Study 2 documented that correlates of cultural and biological forms of essentialism vary across racially dominant White participants and participants from subordinated, non-White racial/ethnic groups. Specifically, endorsing cultural essentialist beliefs positively predicted support for affirmative action and for cultural inclusion, and was unrelated to support for demilitarized border security, among those who identified with subordinated racial categories; these relationships were negative among those who identified with the dominant racial category.

## General discussion

Conventional understandings propose that endorsement of essentialism is negatively related to support for social justice outcomes. A decolonial perspective rooted in the epistemic standpoint of people in marginalized groups suggests otherwise. In particular, results of the present research suggest that conventional understandings are more precisely true of biological essentialism—the belief that racial categories are constituted and defined by underlying biological or genetic characteristics. In line with research that has documented similar correlates of biological essentialism among participants of various racial backgrounds (Williams and Eberhardt, 2008), endorsement of biological essentialism was associated with lower support for social justice policy among participants from both dominant and subordinated identity categories in our study.

Building on previous research on the cultural features associated with racial categories (No et al., 2008; Hong et al., 2009; Morning, 2009), we document that people can also understand racial essentialism in terms of distinct and defining cultural patterns. Our results indicate patterns of relationship for cultural essentialism that diverged from conventional understandings in hypothesized ways. Among participants from dominant identity categories, results for both biological and cultural essentialism were similar, and resembled conventional understandings of essentialism. In contrast, among participants from subordinated identity categories, endorsement of cultural essentialism was more compatible with support for egalitarian policy, especially affirmative action and cultural inclusion.

In general, endorsement of cultural essentialism among participants from subordinated identity categories was positively associated with support for egalitarian policy. This relationship was especially true of support for affirmative action and cultural inclusion, but it did not reach conventional levels of statistical significance for the demilitarization of border security measure. These findings are in line with the proposition that different kinds of essentialism may have divergent implications for policies that refer to targets within (affirmative action and cultural inclusion) vs. outside (demilitarization of borders) one's society. Regardless of racial category, endorsement of cultural essentialism might be associated with doubts about the cultural assimilability of immigrants, and a concern that immigration constitutes a symbolic threat to the dominant cultural order, which might increase support for border policing to protect against this threat. If so, then this might explain why endorsement of cultural essentialism was not positively related to support for demilitarized borders among participants with subordinated identities, unlike support for the other two policies.

One limitation of our studies is that, because of limited sample size, we combined participants who identify with different racial categories under a monolithic “subordinated” category. This categorization overlooks important features of historical engagement with dominant racial communities, which might lead to different implications of essentialism beliefs for different racial groups. An important direction for future work is to investigate the implications of different types of essentialism among people who identify with different racial categories without essentializing various racial groups as a unified category.

Despite its limitations, the conceptual contribution of the current research is to illuminate how endorsement of (cultural) essentialism might afford support for social justice policies, especially among people from marginalized racial communities. Support for social justice policies is often greater among people from subordinated identity categories (i.e., the intended recipients) than people from dominant categories (e.g., Lowery et al., 2006). Results of the present research suggest that this effect is particularly true among people who show moderate to high endorsement of cultural essentialism. This pattern is consistent with the idea that some form of essentialism may be necessary to afford the imagination of community that underlies collective identification, buffers well-being in the face of oppression (Branscombe et al., 1999; Schmitt et al., 2003); and provides a foundation for collective action (e.g., van Zomeren et al., 2008; Haslam and Reicher, 2012). Cultural forms of essentialism may serve this purpose for people from subordinated communities without the negative connotations inherent in biological forms of essentialism, particularly for public support of policies aimed at correcting the social processes that lead to inequality.

## Ethics statement

The Human Research Protection Program of the University of Kansas reviewed and approved the procedures for the studies. Participants read an online information statement and indicated their consent to participate in the studies.

## Author contribution

All authors contributed to the conceptualization of the work, methodological design, statistical analyses and substantial writing. NSY had primary responsibility for data collection and wrote the first draft of the paper.

### Conflict of interest statement

The authors declare that the research was conducted in the absence of any commercial or financial relationships that could be construed as a potential conflict of interest.

## Footnotes

^1^People can also deploy biological essentialist beliefs strategically to achieve recognition or resist oppression. For instance, research indicates that certain aspects of biological essentialist beliefs (e.g., immutability or uncontrollability) attenuate anti-gay (Haslam and Levy, 2006) and anti-fat prejudice (Crandall and Martinez, 1996), and may serve as a basis for identification and collective resistance against the oppression that accompanies such prejudices. In contrast, we propose that, in the specific case of racial identity, cultural (but not biological) essentialist beliefs about racial categories would be strategically deployed among subordinated groups, given the role of biological essentialist beliefs in the history of colonialism and slavery in the US.

^2^All studies reported in this paper conform to ethical standards for research with human subjects, and have been approved by the Human Research Protection Program of the University of Kansas.

^3^Gender (partially) and age information is missing due to an error with data collection.

^4^Because of small sample size, we did not conduct separate correlation analyses among non-White participants. The pattern of results does not change when non-White participants are excluded from the analyses.

^5^Based on an a priori decision, we excluded data from an additional 22 participants who indicated an Asian identity. People of Asian descent often occupy an ambiguous position in the U.S. racial hierarchy—especially with respect to issues of affirmative action—that is difficult to categorize along a binary, dominant-subordinated distinction. Conclusions do not differ if one includes data from Asian participants in the subordinated category.

^6^We use the terms “dominant” and “subordinated” to describe identities in terms of power differentials (privileged versus disadvantaged status). This is a more precise characterization than terms that denote numerical status (e.g., majority or minority), which do not always correspond directly to power differentials (e.g., in South Africa). We use the past participle *subordinated* rather than the adjective *subordinate* to emphasize that the relative status is the result of societal oppression rather than an inherent (or essential) feature of these groups.

^7^We used an online tool to probe all interactions we report (Preacher et al., 2006).
