# Supplementary material for: The (Biological or Cultural) Essence of Essentialism: Implications for Policy Support among Dominant and Subordinated Groups
Source: Front Psychol. 2017 May 30;8:900. doi: 10.3389/fpsyg.2017.00900 (PMC5447748; doi:10.3389/fpsyg.2017.00900)
Supplement: Supplementary file 1 [file DataSheet1.docx]

**APPENDIX**

**Biological Essentialism**

1. I think the chief reason why people of a particular race are so alike in their behavior is that they possess a shared genetic inheritance.
2. I think that differences between people of different races in behavior and personality are largely determined by genetic predisposition.
3. I believe that many talents that individuals of a particular race possess can be attributed to genetic causes.
4. I believe that many differences between humans of different skin color can be attributed to differences in genetic predispositions.

**Cultural Essentialism**

1. You can usually guess the racial community in which a person was raised by knowing what they like and dislike (the kinds of music or films they like, etc.).
2. Growing up in a particular racial community will largely determine how a person behaves in social situations.
3. Different racial groups have characteristically different lifestyles that distinguish them from other racial groups.
4. Being raised in a particular racial community does not determine a person's lifestyle and interests. (R)
5. It would be difficult for a person of one racial group to adopt the way of life of a different racial group.
6. Families tend to raise their children in a way that is characteristic of their racial group.
7. People learn the values and beliefs that are defining characteristics of their racial group.
8. Every racial group has a distinctive, defining culture of their own.
9. Families and communities socialize their children to have the psychological habits that are characteristic of their racial group.
10. Each generation attempts to pass along the cultural traditions associated with their racial group.
11. Across time, different racial groups have developed their own way of understanding the world.
12. People raised in different racial communities learn to behave, think and talk in ways defining of their racial group.

**Support for Affirmative Action**

1. Companies in which racial minorities are underrepresented should try to attract more racial minority applicants.
2. Applicants from minority racial groups should not be given priority over similar applicants from the majority racial group when making hiring decisions for a company position. (R)
3. Companies should implement quotas to make sure racial minorities are represented in their organization.
4. If a White and an African-American applicant for a position have very similar credentials, a company should hire the African-American applicant.
5. Academic institutions should try to reach and attract more students from minority racial groups.
6. Applicants to academic institutions from minority racial groups should not be given priority over similar applicants from the majority racial group. (R)
7. University administrations should implement quotas to make sure racial minorities are represented in the student body.
8. If a White and an African-American applicant to a university have very similar credentials, the university should accept the African-American applicant.

**Support for Cultural Inclusion**

1. The U.S. government should eliminate bilingual education programs in public schools. (R)
2. The U.S. government should eliminate funding for ethnic studies programs in high schools and universities. (R)
3. American businesses, such as supermarkets, should provide the option to print receipts in multiple languages.
4. Educational institutions across the United States should accommodate students who need to take days off for religious observance for all religions.
5. Public institutions should enforce dress codes and not allow people to wear traditional clothes. (R)

**Support for Demilitarized Borders**

1. The U.S. government should increase spending directed at securing the U.S. border. (R)
2. The U.S. government should increase its deployment of National Guard troops to secure the U.S. border. (R)
3. The U.S. government should stop spending money on extending the fence built along the U.S. border.
4. The U.S. government should increase the use of technology-based surveillance systems at the U.S. border, such as aerial surveillance systems. (R)
5. The U.S. government should reduce the number of Border Patrol traffic checkpoints throughout the country.
